# Dry skin and blistering in childhood

**DOI:** 10.1111/ced.12895

**Published:** 2016-09-23

**Authors:** A. Dubois, M. Arefi, M. P. Splitt, S. Leech, S. Natarajan, N. Rajan

**Affiliations:** ^1^ Department of DermatologyRoyal Victoria InfirmaryNewcastle upon TyneUK; ^2^ Institute of Genetic MedicineUniversity of Newcastle upon TyneNewcastle upon TyneUK

## Clinical findings

**Figure 1:**
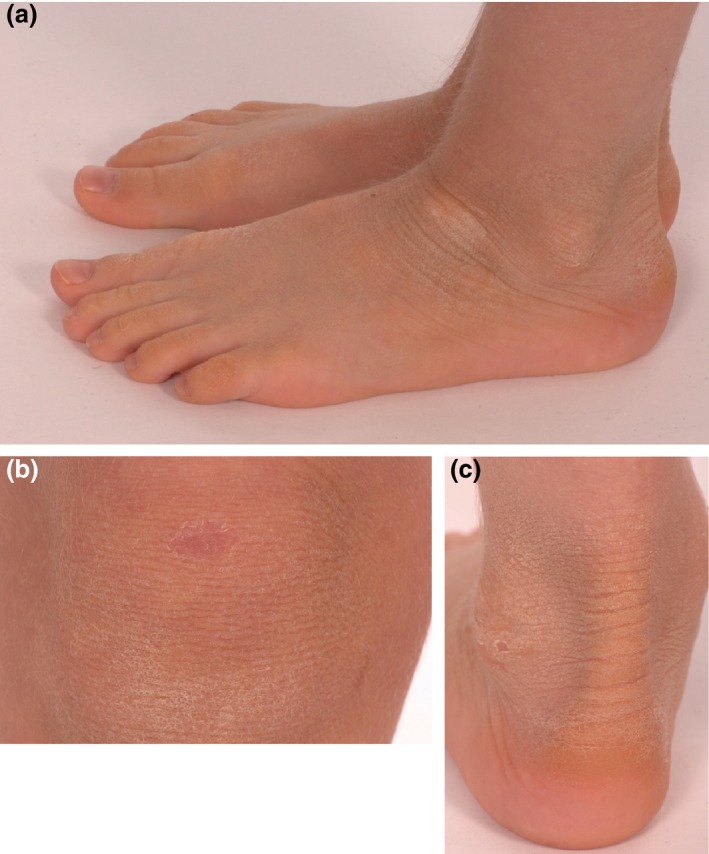
(a) Light‐grey hyperkeratosis overlying the ankle and the dorsa of the feet; (b) superficially denuded area with collarette border on the skin overlying the knee (known as the Mauserung phenomenon, or moulting), which develops due to superficial blistering and shedding of the stratum corneum; (c) hyperkeratosis of the ankle, with well‐defined peeling skin, consistent with the Mauserung phenomenon, i.e. small patches of apparently normal skin in the middle of areas of hyperkeratosis.

A 5‐year‐old boy presented with a history of dry scaly skin. He had been born at term, with no collodion membrane or erythroderma noted at delivery. Skin changes were noted soon after birth, with widespread dryness and occasional blistering, mainly affecting the toes. Previous treatment with emollients and topical corticosteroids had not resulted in improvement. There was no family history of dermatological disease, and his parents were not related. Physical examination revealed mild, light‐grey hyperkeratosis, particularly on the extensor aspects of the skin overlying the joints and the dorsal surfaces of the feet. In addition, superficially denuded areas with collarette‐like borders (known as the Mauserung phenomenon) were seen, most notably on the knees (Fig. 1a–c). The palms, soles, head and neck were spared, and the hair and all nails were normal.

## Histopathological findings

**Figure 2:**
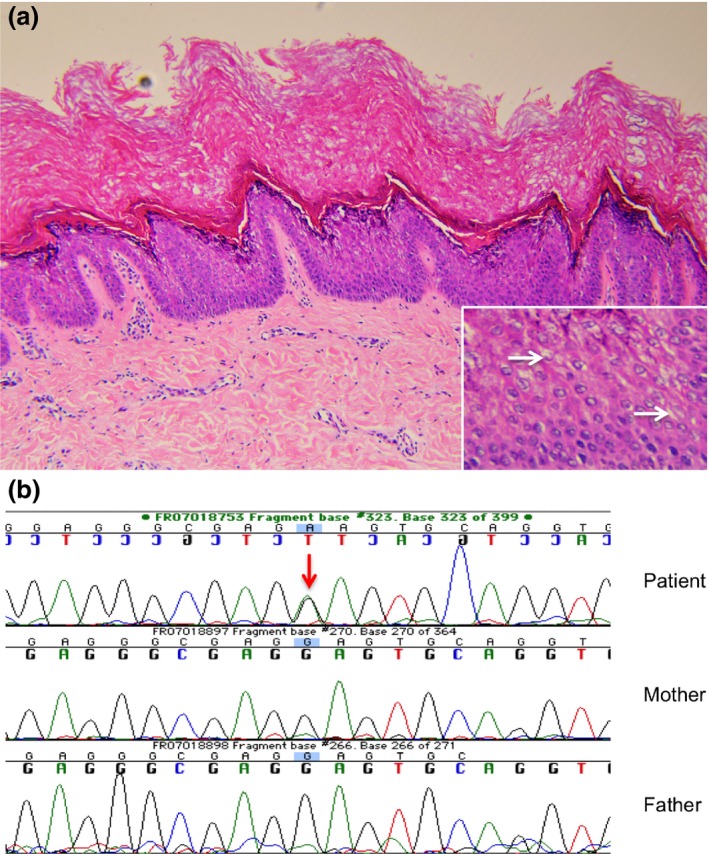
(a) Marked hyperkeratosis and a prominent granular layer, with (inset) scattered vacuolization. Haematoxylin and eosin, original magnification (a) ×* *20; (inset) ×* *40. (b) Recurrent, sporadic mutation in the *KRT2* gene (c.1462G>A, p.E494K; reference sequence: NM_000423.2) demonstrated in the genomic DNA of the affected patient (red arrow), which was not present in the genomic DNA of either parent.

Histological examination of a skin biopsy taken from the ankle region showed marked hyperkeratosis of the stratum corneum and a prominent granular layer with vacuolization (Fig. 2a).

What is your diagnosis?

## Diagnosis

Superficial epidermolytic ichthyosis (SEI) (formerly known as ichthyosis bullosa of Siemens^1^).

## Discussion

Ichthyosis as a general term describes skin changes characterized by hyperkeratosis and/or scaling. The nomenclature of the inherited ichthyoses has been revised recently.^1^ Disorders caused by keratin mutations come under the umbrella term of keratinopathic ichthyoses. Epidermolytic ichthyosis (EI) (formerly known as bullous ichthyosis, bullous congenital ichthyosiform erythroderma, epidermolytic hyperkeratosis or ichthyosis exfoliativa) and SEI are both included within this group.

SEI is an autosomal dominant disorder affecting the keratin (K)2 filament. It was first described by Siemens in 1937, when it was recognized as distinct from the more severe EI, which affects both K1 and K10 filaments. Specific keratins are expressed in different cells of the epidermis; K1 and K10 are expressed in the suprabasal layer, whereas K2 is expressed later in differentiation, in the upper spinous and granular layers.^2^ This expression pattern accounts for the milder phenotype seen in SEI. Unlike EI, SEI does not tend to present with erythroderma at birth, and the skin changes of hyperkeratosis and scaling are often more subtle, commonly affecting the extensor surfaces around joints. However, these disorders can overlap clinically and are then difficult to distinguish by skin changes alone, making DNA sequencing an important diagnostic tool.

Mutations in *KRT2* cause SEI, and since this was elucidated in 1994,^3^ 15 disease‐causing mutations have been reported. A review of the literature revealed no obvious relationship between the mutation and the resulting clinical phenotype in terms of severity, distribution or distinctive features of the condition.

Keratins are intermediate filament proteins. Pairs of type I (acidic) and type II (basic) keratins coil together to form heterodimers, which provide structural integrity to the keratinocytes within the epidermis. Causative mutations in SEI mainly affect the highly conserved 2B helix of the K2 protein, and less commonly the 1A helix, both functionally important for the formation of a heterodimer with its partner keratin.

The findings of hyperkeratosis and blistering in our patient pointed towards a keratinopathic ichthyosis. The distinctive focal skin peeling, together with the mild phenotype and epidermolysis close to the stratum corneum raised the suspicion of SEI. To confirm this clinical diagnosis, sequencing of the *KRT2* gene was performed, which revealed a heterozygous G to A transition in codon 494 (c.1462G>A, p.E494K; reference sequence: NM_000423.2), which has been reported once previously^4^ and is known to be a pathogenic mutation (Fig. 2b). The substitution of lysine for glutamic acid in the K2 protein interferes with heterodimer formation. Subsequent testing of our patient's parents revealed that the mutation had occurred *de novo*.

Treatment for SEI is aimed at symptom relief. Topical therapy with keratolytics and emollients is useful for the hyperkeratosis. Retinoids can also be used, either topically for milder symptoms or orally for more severe cases. The blistering seen in SEI improves with age.

**Learning points**

- SEI is a rare autosomal dominant genodermatosis that can be inherited or occur on a sporadic basis.
- Key features include hyperkeratosis, blistering and localized superficial skin peeling. Blistering improves with age.
- Histologically, epidermolysis is seen in the granular layer.
- Genetic testing for a mutation in *KRT2* can be useful in confirming the diagnosis.
